# Supplementary material for: Complexome profiling of the Chlamydomonas psb28 mutant reveals TEF5 as an early PSII assembly factor
Source: Plant Cell. 2025 Mar 17;37(6):koaf055. doi: 10.1093/plcell/koaf055 (PMC12164590; doi:10.1093/plcell/koaf055)
Supplement: koaf055_Supplementary_Data [file koaf055_supplementary_data.zip › psb28_interactive.zip!/plots/intensity/qualityPlots/peptides/MCP11_Cre06.g295400.t1.2.html]

### Cre06.g295400.t1.2

Trivial name: MCP11  
SeqCoverage [median]: 14.20  
Mass: 34.37 kDa  
  
 

| Sequence | MissedCleavage | Mass [Da] | wtIntensity | muIntensity | Score |
| --- | --- | --- | --- | --- | --- |
| ASLFGAFGASK | 0 | 1054.54 | 395.82 | 0.00 | 76.23 |
| NTPANAAYLGSFEVLK | 0 | 1693.87 | 375.90 | 0.00 | 74.46 |
| SQLQVQIIR | 0 | 1083.64 | 484.94 | 372.91 | 70.92 |
| YTTIPTTAR | 0 | 1022.54 | 1030.58 | 284.33 | 74.92 |
|  |  |  | 2287.2 | 657.2 |  |
